# Situation report: reactions to mammalian amniotic fluid and blood among veterinarians

**DOI:** 10.3389/falgy.2026.1873093

**Published:** 2026-06-26

**Authors:** Maureen Milon, Benjamin Savoye, Cécile Morice, Yann Ollivier, Brigitte Le Mauff, François Ledoyen, Julien Serrier

**Affiliations:** 1 University Center for Allergic Diseases (CUMA), Caen University Hospital, Caen, France; 2 Department of Immunology and Histocompatibility, Caen University Hospital, Caen, France; 3 Physiopathology and Imaging of Neurological Disorders (PhIND), INSERM UMR-S U1237, GIP Cyceron, Caen, France; 4 University of Caen Normandy, Caen, France; 5 GREYC UMR 6072, University of Caen Normandy, ENSICAEN, Caen, France

**Keywords:** amniotic fluid, blood, bovine, occupational exposure, protein contact dermatitis, veterinarians

## Abstract

**Background:**

Reactions to animal biological fluids are poorly described in veterinary occupational health, despite exposures during obstetrical and surgical procedures. This study aimed to characterize the clinical features and risk determinants of reactions to mammalian amniotic fluid (AF) and blood in veterinarians.

**Methods:**

A questionnaire was distributed to veterinarians practicing in France and Belgium. Data collected included demographics, allergic history, types of animal exposure, clinical features of reactions, time of onset of symptoms, use of protective equipment.

**Results:**

Among 317 respondents, 46% reported reactions to AF and 16% to blood. These reactions were predominantly cutaneous and often delayed, contrasting with the more frequent respiratory symptoms reported after common animal contact. Most cases were linked to bovine exposure and occurred early in obstetrical practice. A strong association was observed between the onset of parturitions practice and first symptoms. Increased number of parturitions and absence of protective measures were associated with higher risk.

**Conclusions:**

Reactions to mammalian AF and blood represent a distinct occupational entity in veterinary medicine. The symptoms could be suggestive of protein contact dermatitis. Early exposure and insufficient protection may contribute to their development, highlighting the need for preventive strategies and validated diagnostic tools.

## 1 Introduction

Occupational allergies are a widespread public health issue with long lasting impacts on individuals and work quality of life. Among animal care professionals, veterinarians are particularly exposed to an array of natural allergens, including airborne particles such as pollen, house dust mites and animal dander, as well as cutaneous allergens found in animal secretions and handled products (1, 2). Despite the chronic nature of these exposures, cutaneous allergic diseases in this population remain poorly studied and likely underdiagnosed.

One particular concern is protein contact dermatitis (PCD), a form of contact dermatitis caused by high-molecular-weight proteins. First described in 1976 in food handlers, PCD typically presents as chronic eczema on the hands and forearms, with urticarial or vesicular acute flares after contact with the allergen (3). Diagnosis is based on clinical history and is supported by positive skin prick tests. Management includes allergen avoidance, protective measures, and topical corticosteroids if lesions persist (4). The prevalence of PCD was estimated at 0.33% in a retrospective study conducted by Barbaud et al. in a French dermatology and allergology center. The condition is more frequently observed in occupations with repeated exposures to animal or food proteins such as catering professionals, bakers, farmers, animal care workers, and veterinarians (5). Respiratory symptoms are frequently associated, affecting up to 46% of individuals, with rhinitis in 38% and asthma in 21% of cases. Some allergens, such as bovine epithelium, cereals, and ornamental plants proteins, have been more strongly associated with respiratory involvement (6).

In rural or large-animal veterinary practice, professionals are regularly exposed to protein-rich biological fluids, including AF and blood during parturitions or surgical procedures. This study was therefore designed to better characterize reactions related to contact with mammalian AF and blood among veterinarians.

Our objective was to describe their clinical features and to identify occupational and individual risk factors. To our knowledge, this is the first nationwide study focusing on reactions to these specific biological fluids.

## 2 Materials and methods

### 2.1 Study design and data collection

This observational, descriptive, retrospective, cross-sectional study was carried out from February to November 2023 with the support of French and Belgian Veterinary Orders and Caen University Center for Allergic Diseases.

Data were collected through a digital questionnaire (See Supplementary Data S1) distributed by the French and Belgian Veterinary Orders to all members after approval by their respective scientific board. The questionnaire asked participants about reactions following different types of animal exposures, including mammalian AF, blood, and contact with animals outside of these fluids—such as routine handling, care, and minor procedures—hereafter referred to as “common animal contact.” As all respondents were licensed veterinarians, they were considered healthcare professionals able to identify the clinical symptoms addressed. To support symptom recognition during data collection, photographs of cutaneous symptoms (urticaria and eczema) were included in the questionnaire. Two reminder emails were subsequently sent at two-month intervals. An introductory information note was provided to all participants before completing the questionnaire.

The study protocol was approved by the Caen University Hospital local ethic committee (CLERS, n°2023-4103) and was conducted in accordance with the French data protection guidelines.

### 2.2 Statistical analysis

Descriptive statistics were used to summarize demographic and exposure variables. Continuous variables were presented as means ± standard deviations, and categorical variables as counts and percentages. Missing data for some variables were excluded from the analysis.

An overall *χ*^2^ of independence test was performed to compare the groups, followed by *post hoc* pairwise comparisons using Fisher's exact test. The resulting p-values were adjusted for multiple comparison using the Benjamini-Hochberg false discovery rate test.

To identify risk factors associated with reactions to mammalian AF and blood, separate multivariable mixed-effects binary logistic regression models were fitted. Adjusted odds ratios (Adj. ORs) with 95% confidence interval (CI) were estimated for each variable while controlling for potential confounders, and significance was assessed using Wald tests.

A *p* < 0.05 was considered statistically significant for all analyses.

All statistical and descriptive analyses were performed using Python (version 3.10.12) with the statsmodels (version 0.14.4), scipy (version 1.17.10) and pandas (version 2.2.3) packages. Data visualization was carried out using seaborn (version 0.13.2) and geoplot (version 0.5.1).

## 3 Results

### 3.1 Respondent characteristics

A total of 317 responses were received from 248 French and 69 Belgian veterinarians. The respondents accounted for 1.19% (248/20,844) of veterinarians registered with the French Veterinary Order and 2.38% (69/2,904) of those registered with the Belgian Veterinary order in 2023 (7). The mean age was 42 years, in line with the average for French veterinarians (41 - 44 years). Women accounted for 207 respondents (65%) and men for 110 (35%), compared with 58% and 42% respectively, in the French registered population. For Belgium, no official data were available for comparison in 2023. However, the 2025 OBSVET Atlas of Walloon veterinarians reported similar demographics, with a median age of 46 years and a gender distribution of 56% women and 44% men (8).

### 3.2 Clinical features vary with the type of animal exposure

**Figure 1:**
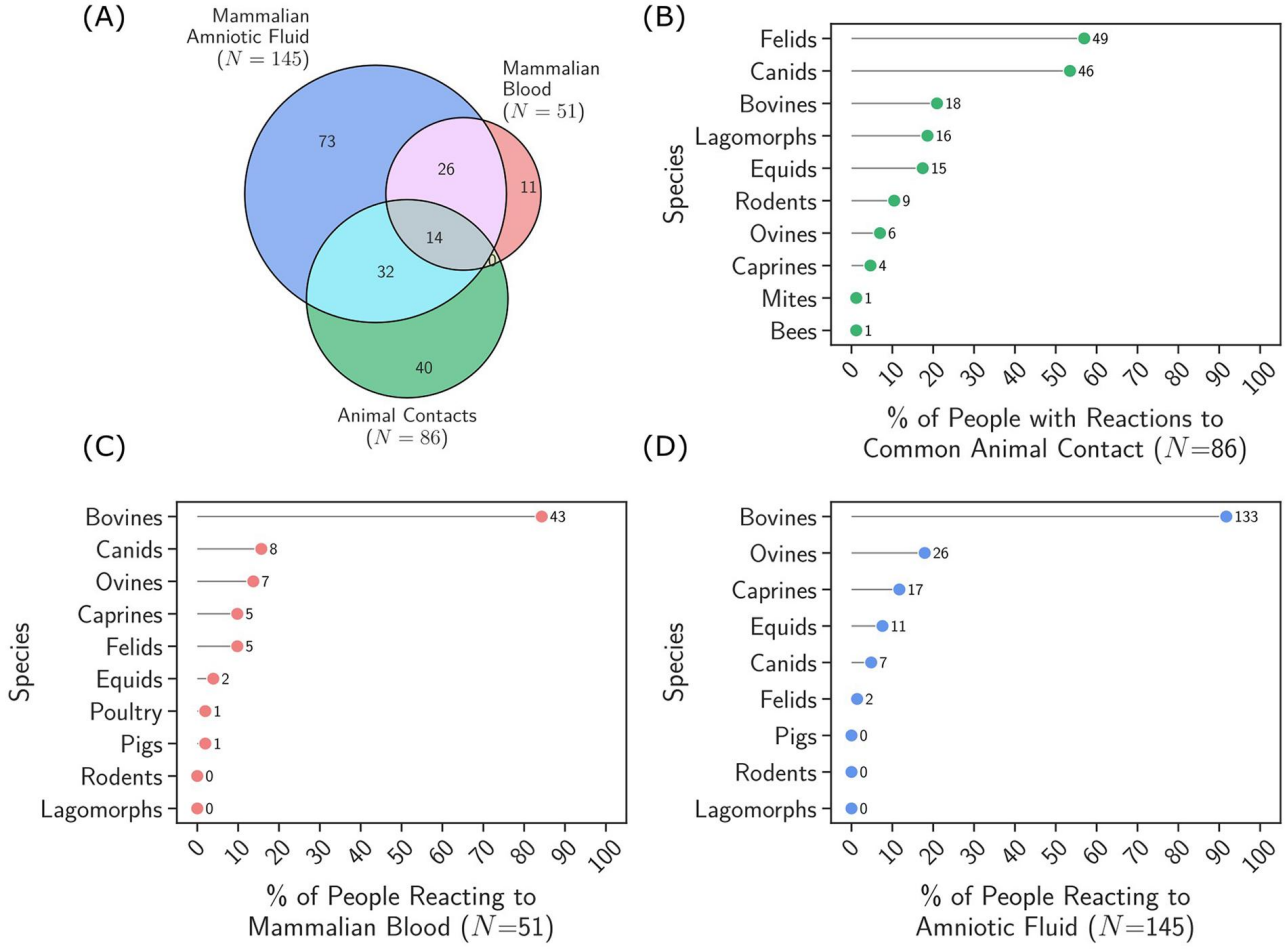
Distribution of self-reported reactions to animal-related exposures in veterinarians. **(A)** Venn diagram showing the overlap of respondents reporting reactions to mammalian amniotic fluid, blood, and common animal contact. **(B)** Top 10 species associated with reactions in veterinarians to mammalian amniotic fluid, **(C)** blood, and **(D)** animal contact.

Out of 317 respondents, 145 (46%) reported reactions to mammalian AF, 51 (16%) to mammalian blood and 86 (27%) to other forms of animal contact. Reactions to more than one exposure were also reported, most frequently involving AF (Figure 1A). Overall, no significant differences were observed between the clinical features of reported AF- and blood-related reactions. Almost all respondents reporting reactions to mammalian AF or blood exhibited cutaneous symptoms after contact (98%), whereas this rate was lower among those reporting reactions after common animal contact (76%, *p* < 0.001). Among these symptoms, eczema was much more frequent during reactions to AF (52%) or blood (53%) than during reactions to common animal contact (7%, *p* < 0.001). Notably, the combination of urticaria followed by eczema was only reported in reactions attributed to AF and blood (20% and 10% respectively).

The most frequent location of skin symptoms was the forearms, hands, and arms, followed by the face, with respective frequencies of 92%, 65%, 37%, 14% for AF-related reactions and 76%, 65%, 24%, 20% for blood-related reactions.

Respiratory symptoms were markedly less common in reactions related to AF or blood exposure (11–12%) compared to those linked to other types of animal contact (71%, *p* < 0.001), where rhinitis (69%), asthma (27%), and conjunctivitis (45%) were frequently reported.

The time of onset of symptoms also differed according to exposures. While most of AF reactions occurred within the first six hours, they were less concentrated in the first hour (51%) than animal contact reactions (84%, *p* < 0.001). A greater proportion appeared later, with 11% after six hours compared with only 3% following common animal contact. A similar pattern was observed with blood exposure, for which 59% of cases occurred beyond the first hour and 6% after 6 h. In addition, the persistence of symptoms was more pronounced after AF and blood exposures, with 31% and 20% of reactions respectively lasting longer than 72 h, whereas none of the animal contact reactions exceeded this duration.

Overall, approximately 40% of respondents who had experienced reactions to biological fluids reported having consulted an allergist (Table 1).

**Table 1:** Clinical characteristics of reactions to amniotic fluid, blood, and animal contact.

Respondent characteristic	Mammalian Amniotic Fluid (AF)	Mammalian Blood (BLD)	Animal Contacts (AC)	*P*-values			
	(*N* = 145)	(*N* = 51)	(*N* = 86)	Overall	AF vs. BLD	AF vs. AC	BLD vs. AC
Cutaneous symptoms							
Cutaneous symptoms – total	142 (98%)	46 (90%)	41 (48%)	*P* < .001***	1.00	*P* < .001***	*P* < .001***
Urticaria	74 (51%)	23 (45%)	35 (41%)	1.00	1.00	1.00	1.00
Eczema	75 (52%)	27 (53%)	6 (7%)	*P* < .001***	1.00	*P* < .001***	*P* < .001***
Urticaria followed by eczema	29 (20%)	5 (10%)	0 (0%)	*P* < .001***	1.00	*P* < .001***	0.19
Edema	17 (12%)	6 (12%)	0 (0%)	0.12	1.00	0.01*	0.07
Cutaneous symptom location							
Hands	94 (65%)	33 (65%)	NA	1.00	1.00	NC	NC
Arms	54 (37%)	12 (24%)	NA	1.00	1.00	NC	NC
Forearms	134 (92%)	39 (76%)	NA	0.16	0.14	NC	NC
Face	21 (14%)	10 (20%)	NA	1.00	1.00	NC	NC
Neck	3 (2%)	0 (0%)	NA	1.00	1.00	NC	NC
Trunk	7 (5%)	0 (0%)	NA	1.00	1.00	NC	NC
Thighs	7 (5%)	2 (4%)	NA	1.00	1.00	NC	NC
Legs	7 (5%)	1 (2%)	NA	1.00	1.00	NC	NC
Feet	2 (1%)	1 (2%)	NA	1.00	1.00	NC	NC
Back	6 (4%)	2 (4%)	NA	1.00	1.00	NC	NC
Respiratory and ocular symptoms							
Respiratory and ocular symptoms – total	26 (18%)	16 (31%)	64 (74%)	*P* < .001***	1.00	*P* < .001***	*P* < .001***
Rhinitis	11 (8%)	5 (10%)	59 (69%)	*P* < .001***	1.00	*P* < .001***	*P* < .001***
Asthma	7 (5%)	1 (2%)	23 (27%)	*P* < .001***	1.00	*P* < .001***	*P* < .01**
Conjunctivitis	22 (15%)	14 (27%)	39 (45%)	*P* < .001***	1.00	*P* < .001***	1.00
Other symptoms							
Collapse	2 (1%)	0 (0%)	NA	1.00	1.00	NC	NC
Delay before first symptom onset							
<1h	74 (51%)	30 (59%)	72 (84%)	*P* < .001***	1.00	*P* < .001***	0.07
1-6h	51 (35%)	18 (35%)	11 (13%)	0.02*	1.00	*P* < .01**	0.08
6-24h	0 (0%)	0 (0%)	2 (2%)	NC	NC	NC	NC
24-72h	15 (10%)	2 (4%)	1 (1%)	0.49	1.00	0.20	1.00
>72h	2 (1%)	1 (2%)	0 (0%)	1.00	1.00	1.00	1.00
Last symptom persistence							
<6h	33 (23%)	20 (39%)	56 (65%)	*P* < .001***	0.88	*P* < .001***	0.13
6-24h	35 (24%)	10 (20%)	16 (19%)	1.00	1.00	1.00	1.00
24-72h	29 (20%)	10 (20%)	14 (16%)	*P* < .01**	1.00	*P* < .001***	*P* < .001***
>72h	45 (31%)	10 (20%)	0 (0%)	*P* < .001***	1.00	*P* < .001***	*P* < .001***
Allergy care							
Allergist consultation	55 (38%)	19 (37%)	43 (50%)	1.00	1.00	1.00	1.00

NA, not asked in the questionnaire; NC, not calculated.

* *P* < 0.05.

** *P* < 0.01.

*** *P* < 0.001.

These data suggest a clinical profile for reactions involving mammalian AF or blood, characterized by predominant skin involvement and a prolonged course, as opposed to the mainly respiratory and short-lived reactions reported after other types of animal exposure.

### 3.3 Conditions of mammalian AF and blood exposure

Bovines were by far the most frequently implicated species in reactions to mammalian AF and blood, cited by 91.7% and 84.3% of respondents respectively. Other species mentioned in biological fluid-related reactions included, in decreasing order of frequency, livestock such as ovines and caprines, followed by equids. In contrast, felids and canids, who ranked first (57%) and second (54%) among species associated with common animal exposure reactions were much less frequently mentioned to trigger reactions to biological fluids. These two species were estimated to trigger only 6.2% of AF-related reactions and 26% of those involving blood (Figures 1B–D).

**Figure 2:**
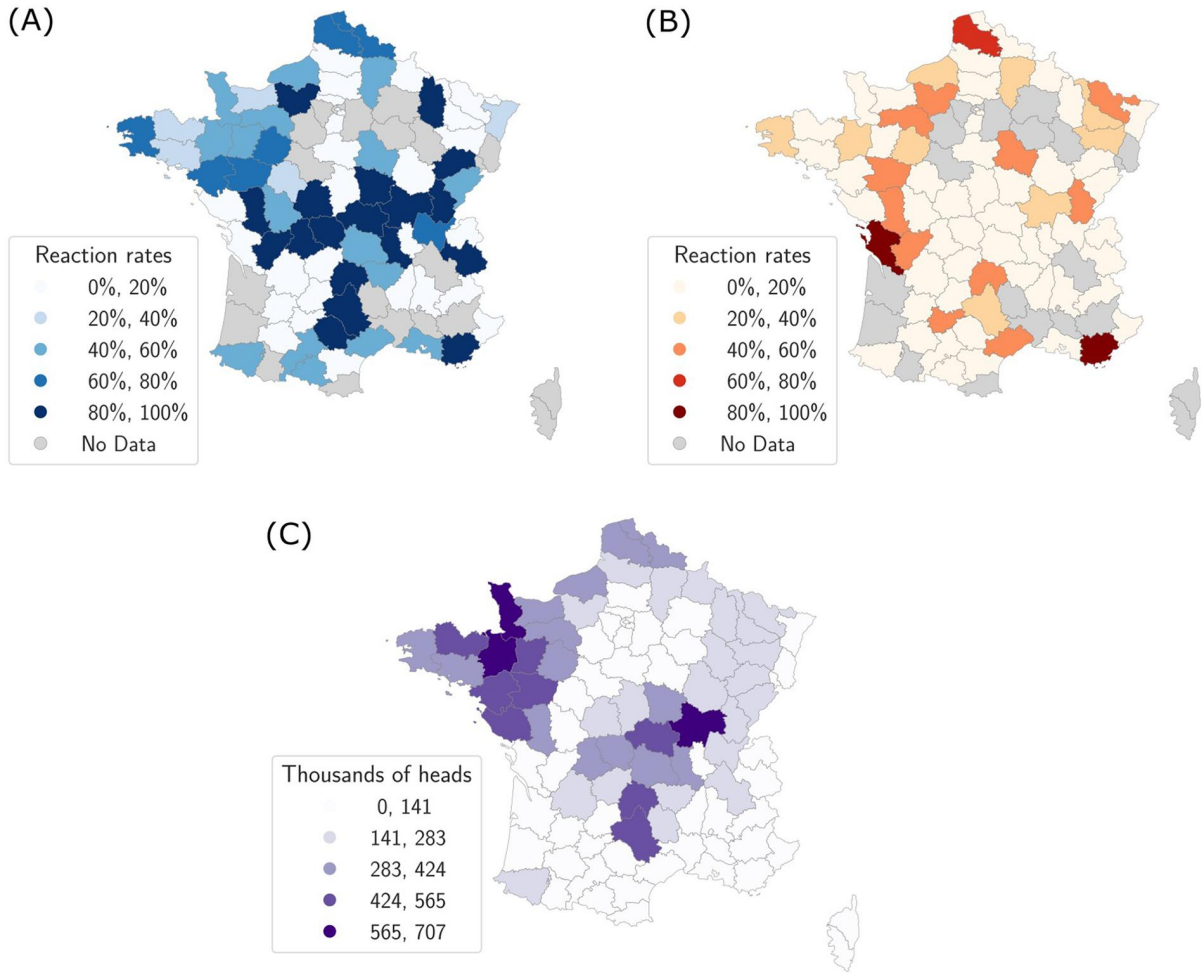
Geographic distribution of veterinarian reactions to **(A)** mammalian amniotic fluid and **(B)** blood, compared to **(C)** the distribution of cattle in France in 2023 [INSEE data (9)].

The geographical distribution of French veterinarians reporting reactions was compared to that of the bovine livestock in France. An overlap was observed for AF-related reactions, primarily in the rural regions of northern, central and western France, but was less pronounced for blood-related reactions (Figure 2). The same kind of analysis could not be performed with Belgian respondents since their geographical distribution was not collected in the questionnaire.

**Figure 3:**
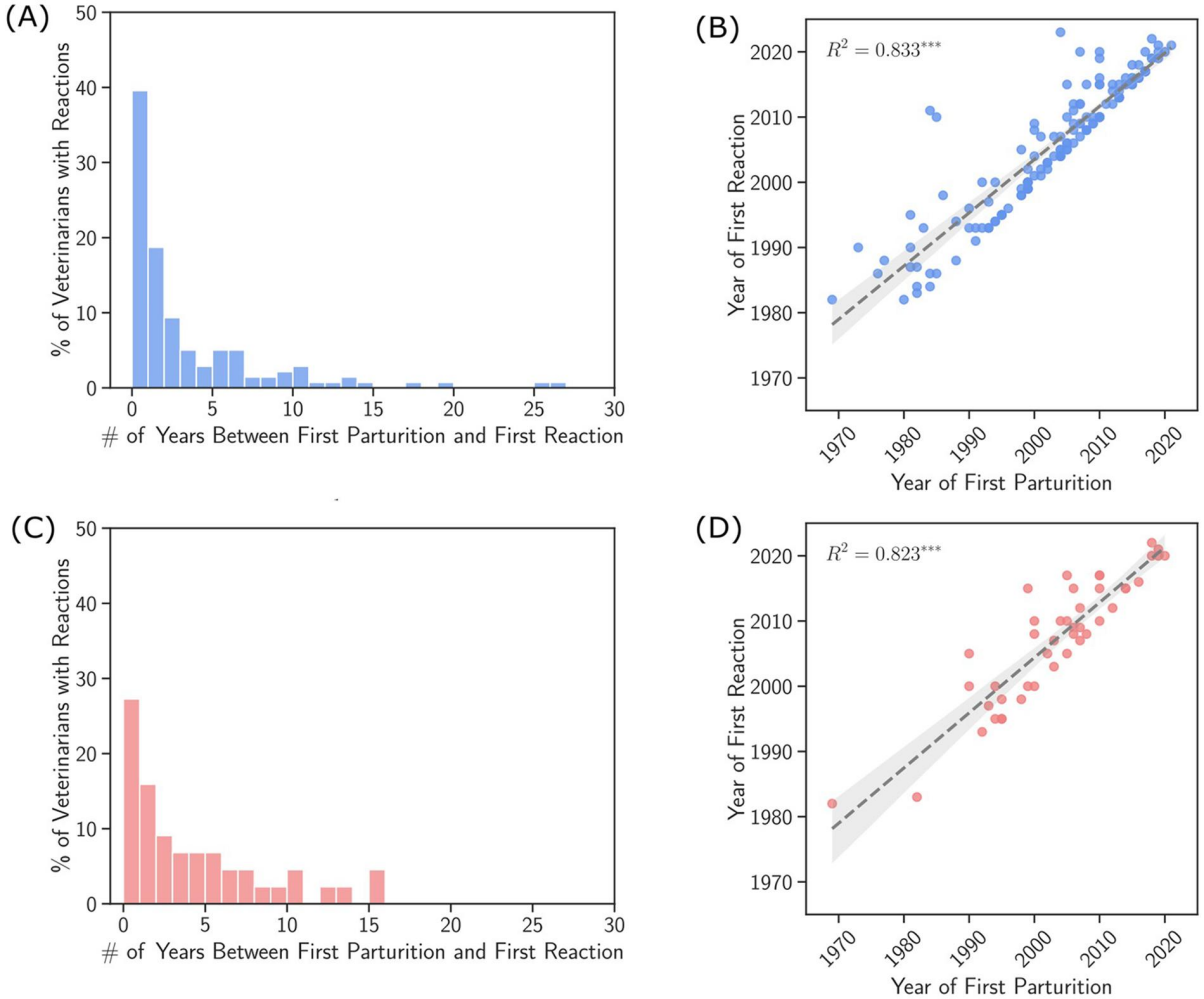
Association between parturition and veterinarians’ reactions to **(A,B)** mammalian amniotic fluid and **(C,D)** blood. *** *p* < 0.001.

In terms of timing, reactions to mammalian AF and blood were often reported during the initial years in which veterinarians began performing parturitions. The mean interval between initial exposure to AF and symptom onset was 3 years, compared with 4 years for blood exposure. A strong correlation was observed between the year of first parturition and the year of first reaction, both for AF (R^2^ = 0.833, *p* < 0.001) and for blood (R^2^ = 0.823, *p* ≤ 0.001, Figure 3). These observations support the hypothesis that bovine exposure and early obstetrical activities are key contextual factors in the development of occupational reactions to mammalian biological fluids.

### 3.4 Risk and protective factors for reactions to AF and blood

Risk factors associated with reported reactions to mammalian AF and blood were evaluated using multivariable logistic regression (Table 2). A dose-response relationship was observed between the number of parturitions and the risk of reaction: veterinarians with 100–1,000 parturitions had an OR of 4.07 (2.10–7.91, *p* < 0.001), those with 1,000–3,000 parturitions an OR of 5.94 (2.21–15.95, *p* < 0.001), and those with over 3,000 parturitions an OR of 2.89 (1.02–8.16, *p* = 0.05), compared to the reference group (<100 parturitions). Prior reactions to blood were associated with reactions to AF (OR 4.9, 2.18–10.98, *p* < 0.001). Previous allergic reactions to other animal exposure were also associated with a higher risk of reaction (OR 2.32, 1.13–4.78, *p* = 0.02). No association with cutaneous atopy was notices for reactions to AF (OR 4.41, 0.79–24.59, *p* = 0.09). Lack of protective measures during deliveries significantly increased risk (OR 4.12, 1.22–13.88, *p* = 0.02). Among protective equipment, the most frequently worn were surgical gowns (63%), latex gloves (70%), and vinyl gloves (28%). The use of vinyl gloves was associated with higher odds of reactions (OR 2.07, 1.08–3.93, *p* = 0.03). Interestingly, a history of tick bites was negatively associated with AF reactions (OR 0.40, 0.22–0.72, *p* < 0.01) but not for blood reactions.

**Table 2:** Risk factors for mammalian amniotic fluid and blood among reported cases.

Respondent characteristic	Reactions to mammalian amniotic fluid				Reactions to mammalian blood			
	Yes (%) (*N* = 145)	No (%) (*N* = 172)	Adj. OR (95% CI)	*P*-value	Yes (%) (*N* = 51)	No (%) (*N* = 266)	Adj. OR (95% CI)	*P*-value
Age	44 (*±*11)	41 (*±*13)	1.0 (0.98–1.03)	0.80	44 (*±*9)	42 (*±*12)	1.01 (0.97–1.04)	0.74
Sex								
Female	82 (57%)	125 (73%)	*Reference*	–	27 (53%)	180 (68%)	*Reference*	–
Male	63 (43%)	47 (27%)	1.26 (0.64–2.47)	0.50	24 (47%)	86 (32%)	1.31 (0.56–3.03)	0.53
Number of parturitions								
0–100	27 (19%)	94 (55%)	*Reference*	–	9 (18%)	112 (42%)	*Reference*	–
100–1,000	63 (43%)	48 (28%)	4.07 (2.1–7.91)	*P* < .001***	19 (37%)	92 (35%)	2.1 (0.8–5.52)	0.13
1,000–3,000	33 (23%)	12 (7%)	5.94 (2.21–15.95)	*P* < .001***	16 (31%)	29 (11%)	5.23 (1.57–17.37)	0.01**
>3,000	22 (15%)	18 (10%)	2.89 (1.02–8.16)	0.05*	7 (14%)	33 (12%)	1.76 (0.46–6.81)	0.41
Other reactions								
Other animal contact	46 (32%)	40 (23%)	2.32 (1.13–4.78)	0.02*	14 (27%)	72 (27%)	0.69 (0.27–1.72)	0.43
Mammalian amniotic fluid	–	–	–	–	40 (78%)	105 (39%)	4.84 (2.15–10.87)	*P* < .001***
Mammalian blood	40 (28%)	11 (6%)	4.9 (2.18–10.98)	*P* < .001***	–	–	–	–
Protections equipment	16 (11%)	10 (6%)	1.79 (0.66–4.86)	0.25	8 (16%)	18 (7%)	2.47 (0.87–7.02)	0.09
Allergy predisposition								
Food allergy	21 (14%)	19 (11%)	1.16 (0.5–2.67)	0.73	9 (18%)	31 (12%)	1.31 (0.46–3.71)	0.61
Cutaneous atopy	9 (6%)	3 (2%)	4.41 (0.79–24.59)	0.09	4 (8%)	8 (3%)	1.09 (0.24–5.03)	0.91
Respiratory allergy	55 (38%)	68 (40%)	0.59 (0.31–1.14)	0.12	21 (41%)	102 (38%)	1.3 (0.57–2.96)	0.53
Protective equipment used during parturitions								
No protections	13 (9%)	6 (3%)	4.12 (1.22–13.88)	0.02*	1 (2%)	18 (7%)	0.14 (0.01–1.37)	0.09
Surgical gown	108 (74%)	109 (63%)	1.3 (0.68–2.5)	0.43	39 (76%)	178 (67%)	0.73 (0.3–1.78)	0.49
Polypropylene gloves	19 (13%)	9 (5%)	2.3 (0.87–6.05)	0.09	4 (8%)	24 (9%)	0.42 (0.12–1.48)	0.18
Latex gloves	77 (53%)	121 (70%)	0.8 (0.45–1.42)	0.44	30 (59%)	168 (63%)	1.33 (0.64–2.79)	0.45
Nitrile gloves	29 (20%)	32 (19%)	0.95 (0.46–1.96)	0.88	13 (25%)	48 (18%)	1.21 (0.5–2.95)	0.67
Vinyl gloves	41 (28%)	32 (19%)	2.07 (1.08–3.93)	0.03*	9 (18%)	64 (24%)	0.34 (0.14–0.85)	0.02*
Other variables								
Tick bite	87 (60%)	131 (76%)	0.4 (0.22–0.72)	*P* < .01**	31 (61%)	187 (70%)	0.68 (0.33–1.42)	0.31

ORs are derived from the coefficients of a multivariable binomial logistic regression model. Percentages are calculated within each outcome group (“reaction” or “no reaction”).

* *p* < 0.05.

** *p* < 0.01.

*** *p* < 0.001.

Adj. OR, Adjusted Odds Ratio; CI, Confidence Interval.

For reactions to mammalian blood, the number of parturitions also increased the risk, with 1,000–3,000 parturitions associated with a fivefold increase in odds ratio (OR 5.23, 1.57–17.37, *p* = 0.01). Reactions to both AF and blood were observed in 40 respondents, corresponding to 78% of those reporting blood reactions and 28% of those reporting AF reactions. Therefore, prior reactions to AF were strongly associated with reactions to blood (OR 4.84, 2.15–10.87, *p* < 0.001). The effect of protective equipment used was similar to that observed among respondents who experienced reactions to AF except for vinyl gloves which appeared protective for blood reactions (OR 0.34, 0.14–0.85, *p* = 0.02) whereas other protective gear showed no significant effect.

These results underline the role of cumulative exposure through parturitions and prior animal-related reactions as risk factors, while the use of protective equipment could attenuate the risk.

## 4 Discussion

This study investigated an occupational health issue affecting veterinarians. It is the first to describe, in a large cohort, reactions occurring after exposures to mammalian AF or blood. Analysis of 317 questionnaire responses from veterinarians in France and Belgium revealed a distinct clinical profile compared to responses associated with common animal contact. In addition, specific occupational risk and protective factors were identified.

Although reactions to AF have been sporadically reported for over four decades, they remain insufficiently documented. Most published data consist of isolated case reports or small series, often lacking specific allergy investigation. A review of the literature reveals fewer than 50 suspected cases of reaction related to obstetrical procedure, with approximately half supported by positive skin tests or specific IgE, mostly to AF (5, 10–15). Occupational reactions related to blood exposure have been primarily studied in the context of slaughterhouse work (16, 17). In our study, nearly half of respondents reported reactions to AF and over 15% to mammalian blood. Even if this high estimated prevalence may be overestimated because respondents with symptoms are more likely to participate in voluntary surveys, it suggests that reactivity to these biological fluids is a relevant concern in veterinary occupational context. Reactions to AF and blood were mainly characterized by skin symptoms, with contact urticaria followed in several cases by eczema-like lesions that could become chronic. This biphasic pattern is compatible with features previously described in PCD (5). The preferential localization of skin lesions on exposed areas, particularly the forearms, hands, and arms, further supports this hypothesis. In contrast, reactions related to common contact with animals were mainly respiratory, likely triggered by common pneumallergens such as saliva and urine found on the skin appendages (18).

Variations in symptom onset and duration could offer insights into the underlying immunopathological mechanisms. Reactions associated with common animal contact were predominantly rapid in onset and short in duration consistent with the clinical presentation of immediate type I hypersensitivity mediated by IgE. In contrast, reactions following exposures to AF and blood sometimes demonstrated a two phases pattern with immediate type I-like reaction as urticaria completed by a more delayed onset of eczema lesions with prolonged course, which could be in line with type IV hypersensitivity. This reaction kinetics appears broadly consistent with descriptions of PCD involving both immediate and delayed mechanisms (19).

The predominance of bovines as the main species involved in reactions to mammalian AF and blood aligns with the high frequency of obstetrical procedures in cattle. Calving assistance is a common veterinary practice in both dairy and beef herds and is more frequent than in other species such as ovines and caprines (20). These interventions produce direct and repeated exposures to large volumes of biological fluids. Moreover, the duration and type of contact with AF differ substantially between large and small animal practices and may explain the overrepresentation of large animal species among those responsible for reported reactions.

Notably, the first reactions to AF and blood appeared to occur predominantly during the early years of performing parturitions, with a strong correlation with the first year of obstetrical practice. By way of comparison, in a study of various types of protein exposures, Hernández-Bel et al. described latency periods for PCD ranging from 2 months to 27 years in a series of 27 (21). Overall, current understanding suggests that sensitization arises more frequently in individuals with impaired skin barrier function such as those with a predisposition to atopic dermatitis, which was not observed in our study (5).

The geographical overlap between reported cases of AF reactions and regions with high cattle density further supports the link between exposure intensity and reactivity risk. For blood-related reactions, the geographical overlap was less clear, maybe reflecting more diverse exposure scenarios. Significant blood contact can occur during major surgical procedures such as laparotomies in cesarean sections and omentopexy in abomasum displacement surgery, which are often performed on cattle. Veterinarians may sometimes perform these interventions outside standard operating room conditions, for example at the farm, increasing exposure likelihood. However, as reactions to AF emerged as a risk factor for blood-related reactions, obstetric procedures could be the main route of sensitization. This is also consistent with the low use of protective equipment during these procedures as Hanzen et al. reported in 2011 that only 34% of european veterinarians used gloves during caesarean sections (22).

As expected, the number of parturitions was identified as a risk factor for reactions to both AF and blood with a clearer dose-response relationship observed for AF. However, this variable was self-reported and may have been under- or overestimated. Furthermore, this could be an indicator of other confounding exposures such as cumulative use of antiseptics, glove materials or antibiotics used as prophylaxis. Such exposures cannot be excluded as contributing factors to the high rate of cutaneous symptoms observed among respondents reporting reactions to AF (98%) or blood (90%), through irritant or allergy contact dermatitis mechanisms, although reactions to protective equipment were not identified as independent predictors.

While the absence of protective equipment was identified as a risk factor, vinyl gloves were associated with a higher risk of reactions to AF, yet appeared protective in cases of blood exposure. This discrepancy could stem from differences in working conditions. Obstetrical procedures involve prolonged contact with biological fluids and repeated friction which can compromise the integrity of vinyl gloves. Compared to latex and nitrile, vinyl gloves have reduced barrier performance when stretched, making them more prone to tearing and leakage (23). In contrast, blood exposure in surgical settings is typically more controlled and involves less mechanical stress, which may allow vinyl gloves to provide efficient protection. Incidentally, it can be speculated that misattribution of biological fluid-related skin reactions to latex allergy may lead some veterinarians with hand dermatitis to switch to fewer protective gloves.

The molecular composition of bovine AF may help explain why reactions to this substance sometimes differed from those triggered by blood. This fluid contains fetal proteins such as fetuin (alpha-2-HS-glycoprotein) and alpha-fetoprotein, along with extracellular matrix components including osteonectin, fibronectin, and collagen precursors which are likely absent from blood or present only at much lower concentrations (24).

A potential link between galactose-alpha-1.3-galactose (alpha-gal) sensitization and reactions to AF was previously suggested by Nuñez-Orjales et al. (25). However, alpha-gal sensitization is mostly triggered by tick bites and our data showed that individuals who reported prior tick bites were actually less likely to experience such reactions. This unexpected protective association argues against alpha-Gal being the primary antigen involved.

A limitation of the study is that the distribution of veterinarians working exclusively or partially in large and/or small animal practice, as well as the time elapsed since graduation, were not collected. These factors could potentially influence both the prevalence of reactions and attitudes toward the use of protective equipment. Notably, despite the frequency of these reactions, few respondents had undergone allergy evaluation, and the results of these assessments were not collected. At the end of the questionnaire, participants were invited to contact the study team if they wished to complete diagnostic investigations but no request was received. In France and Belgium, most veterinarians work in private practice, where health insurance may not automatically cover occupational disease. Moreover, applications for formal recognition are often initiated during career transitions. Veterinarians still actively engaged in their practice may be less inclined to seek formal recognition, which could raise concerns about the sustainability of their professional activity.

## 5 Conclusion

This study suggested a possibly underestimated impact of AF and blood exposure in veterinarians. Improving protective strategies, particularly during obstetrical practice is essential. Affected veterinarians should have access to a full diagnostic evaluation, which should also address potential reactions to exogenous substances used during these procedures such as antiseptics or antibiotics. Given the challenges of using raw biological materials in skin testing and the current lack of standardized tools, *in vitro* investigations such as basophil activation and lymphocyte transformation tests may represent interesting options to explore these kinds of reactions. Improvement of veterinarian information could enhance prevention, care, and reporting. Finally, future immunological studies using IgE immunoblotting and mass spectrometry with sera from sensitized patients would help characterize the responsible allergens, which would subsequently be validated through skin prick or patch tests.

## Data Availability

The raw data supporting the conclusions of this article will be made available by the authors, without undue reservation.
