# Supplementary material for: Situation report: reactions to mammalian amniotic fluid and blood among veterinarians
Source: Front Allergy. 2026 Jun 26;7:1873093. doi: 10.3389/falgy.2026.1873093 (PMC13349834; doi:10.3389/falgy.2026.1873093)
Supplement: Supplementary file 1 [file Datasheet1.docx]

**Data S1 – Questionnaire sent to participants**

English version, translated from French. The original French version follows.

**❖ Questions:**

• Year of birth
• Sex: M/F
• Department (of professional activity)

**❖ DEFINITIONS:**

**URTICARIA** = erythematous, papular eruption resembling nettle stings; transient, migratory and pruritic (Photo provided with the questionnaire)
**ECZEMA** = erythematous, vesicular lesions with irregular borders, pruritic, finely scaly (Photo provided with the questionnaire)

**❖ Year of first parturition**

**❖ Number of parturitions performed since:**

0–100 / 100–1000 / 1000–3000 / >3000

**❖ Have you ever experienced a reaction upon contact with amniotic fluid? YES / NO**

**If yes:**
➢ Year of first symptoms upon exposure to amniotic fluid
➢ In contact with: bovines, equids, ovines, caprines, pigs, lagomorphs, felids, canids, rodents, others
➢ What symptom(s) did you experience?
■ Urticaria
■ Eczema
■ Urticaria progressing to eczema
■ Edema
■ Rhinitis
■ Conjunctivitis
■ Respiratory discomfort
■ Malaise
➢ Time to symptom onset after exposure to amniotic fluid:
<1 hour / 1–6 h / 6–24 h / 24–72 h / >72 h
➢ Duration of symptoms:
<6 h / 6–24 h / 24–72 h / >72 h
➢ Location of eczema/urticaria/edema:
hands / forearms / arms / face / neck / torso / back / feet / legs / thighs

**❖ Have you ever experienced a reaction upon contact with animal blood? YES / NO**

**If yes:**
➢ In contact with: bovines, equids, ovines, caprines, pigs, lagomorphs, felids, canids, rodents, others ➢ What symptom(s) did you experience?
■ Urticaria: localized / generalized
■ Eczema
■ Urticaria progressing to eczema
■ Edema
■ Rhinitis
■ Conjunctivitis
■ Respiratory discomfort
■ Malaise
➢ Time to symptom onset after exposure:
<1 hour / 1–6 h / 6–24 h / 24–72 h / >72 h
➢ Duration of symptoms:
<6 h / 6–24 h / 24–72 h / >72 h
➢ Location of eczema/urticaria/edema:
hands / forearms / arms / face / neck / torso / back / feet / legs / thighs

**❖ Do you have other allergies when in proximity to animals? YES / NO**

**If yes:**
➢ To which components: hair, saliva, urine, others
➢ In contact with: bovines, equids, ovines, caprines, pigs, lagomorphs, felids, canids, rodents, others
➢ Symptoms: asthma, rhinitis, conjunctivitis, urticaria, others
➢ Onset time: <1 h, 1–6 h, 6–24 h, >24 h
➢ Duration: <6 h, 6–24 h, >24 h

**❖ What type of protective equipment do you wear during surgeries and parturitions?**

Surgical gown, nitrile gloves, vinyl gloves, latex gloves, protective goggles, others

**❖ Have you experienced any reactions upon contact with your protective equipment? YES / NO**

**If yes:**
➢ With which protection(s): surgical gown, nitrile gloves, vinyl gloves, latex gloves, protective goggles, others
➢ What symptom(s) did you experience?
■ Urticaria: localized / generalized
■ Eczema
■ Urticaria progressing to eczema
■ Edema
■ Rhinitis
■ Conjunctivitis
■ Respiratory discomfort
■ Malaise
➢ Time to symptom onset after exposure: <1 h / 1–6 h / 6–24 h / 24–72 h / >72 h
➢ Duration of symptoms: <6 h / 6–24 h / 24–72 h / >72 h

**❖ Have you undergone patch testing? YES / NO**

➢ Details of positive tests (if applicable)
➢ Do you have a latex allergy? Positive prick test, positive patch test, positive specific IgE, not applicable

**❖ Do you have respiratory allergy symptoms? Asthma / rhinitis / conjunctivitis: YES / NO**

**If yes:**
➢ Which type(s): asthma / rhinitis / conjunctivitis
➢ Allergen categories: dust mites, pollens, animal dander, molds/spores, others

**❖ Do you have any food allergies? YES / NO**

**If yes:**
➢ When consuming: mammalian meat / mammalian offal / milk or dairy products / other foods (excluding meat products)
➢ From: cattle, goats, sheep, pigs, horses, lagomorphs
➢ Time to symptom onset after ingestion:
<1 h / 1–6 h / 6–24 h
➢ Duration of symptoms: <6 h / 6–24 h / >24 h

**❖ Have you ever been bitten by a tick? YES / NO**

➢ If yes, year of the most recent bite:

We thank you in advance for taking the time to respond to this survey.
If you would like more information or if you have experienced reactions upon contact with amniotic fluid and/or mammalian blood and would like further clinical investigations, please do not hesitate to contact us: “mail address of the authors”

**Questionnaire sent to participants – Original french version**

**❖ Questions à réponses obligatoires :**

- Année de naissance
- Sexe : H/F
- Département (d'exercice professionnel)

**❖ DÉFINITIONS :**

URTICAIRE = éruption érythémateuse, papuleuse, semblable à des piqûres d’orties, fugaces, migratrices et prurigineuses (PHOTO fournies lors de l’envoi du questionnaire)

ECZÉMA = lésion érythémateuse, vésiculeuses, à contour émietté, prurigineuse, finement squameuse (PHOTO fournies lors de l’envoi du questionnaire)

**❖ Année de la première parturition**

**❖ Nombre de parturitions effectuées depuis :** 0-100/100-1000 /1000-3000/>3000

**❖ Avez-vous déjà présenté une réaction au contact du liquide amniotique ?** OUI/NON

**❖ Si oui :**

➢ Année des premiers symptômes au contact du liquide amniotique

➢ Avec : bovins, équidés, caprins, ovins, porcins, lagomorphes, félidés, canidés, rongeurs, autres.

➢ Quel(s) symptôme(s) ?

■ Urticaire

■ Eczéma

■ Urticaire puis évolution vers un eczéma

■ Œdème

■ Rhinite

■ Conjonctivite

■ Gêne respiratoire

■ Malaise

➢ Délai d’apparition par rapport à l’exposition au liquide amniotique ? < 1 heure/ 1-6h/ 6-24h/ 24-72h/>72h

➢ Persistance des symptômes ? < 6h/ 6-24h/ 24-72h/ >72h

➢ Localisation pour eczéma/urticaire/œdème : mains/avant-bras/bras/Visage/cou/tronc/dos/pieds/jambes/cuisses

❖ **Avez-vous déjà présenté des réactions lors du contact avec le sang d’un animal ?** OUI / NON

➢ Si oui, avec : bovin, équidés, caprins, ovins, porcins, lagomorphes, félidés, canidés, rongeurs, autres.

➢ Quel(s) symptôme(s) :

■ Urticaire : localisée/généralisée

■ Eczéma

■ Urticaire puis évolution vers un eczéma

■ Œdème

■ Rhinite

■ Conjonctivite

■ Gêne respiratoire

■ Malaise

➢ Délai d’apparition par rapport à l’exposition au liquide amniotique ? < 1 heure/ 1-6h/ 6-24h/ 24-72h/>72h

➢ Persistance des symptômes ? < 6h/ 6-24h/ 24-72h/ >72h

➢ Localisation pour eczéma/urticaire/œdème : mains/avant- bras/bras/Visage/cou/tronc/dos/pieds/jambes/cuisses

❖ **Avez-vous d’autres allergies à proximité des animaux ?** OUI/NON

**❖ Si oui :**

➢ Avec quel(s) composant(s) : poils, salive, urine, autre

➢ Avec : bovin, équidés, caprins, ovins, porcins, lagomorphes, félidés, canidés, rongeurs, autres.

➢ Symptômes : asthme, rhinite, conjonctivite, urticaire, autre

➢ Délai : <1h, 1-6h, 6-24h, >24h

➢ Durée : <6h, 6-24h, >24h

❖ **Quel type de protection portez-vous lors des chirurgies et des parturitions ? Casaque chirurgicale, gant nitrile, gant vinyle, gant latex, lunettes de protections, autres**

❖ **Réaction au contact de vos protections ?** OUI/NON

**❖ Si oui,**

➢ Avec quelle(s) protection(s) : Casaque chirurgicale, gant nitrile, gant vinyle, gant latex, lunettes de protections, autres

➢ Quels sont les symptômes :

■ Urticaire : localisée/généralisée

■ Eczéma

■ Urticaire puis évolution vers un eczéma

■ Œdème

■ Rhinite

■ Conjonctivite

■ Gêne respiratoire

■ Malaise

➢ Délai d’apparition par rapport à l’exposition au liquide amniotique ? < 1 heure / 1-6h/ 6-24h/ 24-72h/>72h

➢ Persistance des symptômes ? < 6h/ 6-24h/ 24-72h/ >72h

❖ **Avez-vous eu des tests épicutanés = patchs tests ?** OUI/NON

➢ Détail tests positifs

➢ Avez-vous une allergie au latex ? Prick test positif, patch test positif, IgE positive, non concerné

❖ **Avez-vous des symptômes d’allergie respiratoire ?** **asthme/rhinite/conjonctivite :** OUI/NON

➢ Si oui, quel type : asthme/rhinite/conjonctivite

➢ Si oui, quelle(s) catégorie(s) : acariens, pollens, phanères d’animaux, spores/moisissures, autres

❖ **Avez-vous des allergies alimentaires :** OUI/NON

❖ **Si oui :**

➢ En consommant de la viande de mammifère/des abats de mammifère/du lait ou des produits laitiers/autres aliments (hors produits carnés)

➢ Provenant de : bovins, caprins, ovins, porcins, équidés, lagomorphes

➢ Délai d’apparition par rapport à l’ingestion ? < 1 heure / 1-6h/ 6-24h/

➢ Persistance des symptômes ? < 6h/ 6-24h/>24h

**❖ Avez-vous déjà été mordu par une tique ?** OUI/NON

➢ Si oui, année de la dernière morsure ?

Nous vous remercions par avance d’avoir accepté de répondre à cette enquête.

Si vous souhaitez avoir plus d’informations ou si vous présentez des réactions au contact du liquide amniotique et/ou du sang de mammifères et que vous souhaitez bénéficier d’explorations complémentaires, vous pouvez nous contacter : « adresse mail des auteurs »
